# Ecological influence by colonization of fluoride-resistant *Streptococcus mutans* in oral biofilm

**DOI:** 10.3389/fcimb.2022.1106392

**Published:** 2023-01-09

**Authors:** Yan Shen, Fangzheng Yu, Lili Qiu, Mengjia Gao, Puxin Xu, Lingjun Zhang, Xiangyan Liao, Min Wang, Xiangyu Hu, Yan Sun, Yihuai Pan

**Affiliations:** School and Hospital of Stomatology, Wenzhou Medical University, Wenzhou, China

**Keywords:** fluoride-resistant strain, *Streptococcus mutans*, oral biofilm, biofilm shift, cariogenic virulence, dental caries

## Abstract

**Background:**

Dental caries is one of the oldest and most common infections in humans. Improved oral hygiene practices and the presence of fluoride in dentifrices and mouth rinses have greatly reduced the prevalence of dental caries. However, increased fluoride resistance in microbial communities is concerning. Here, we studied the effect of fluoride-resistant Streptococcus mutans (S. mutans) on oral microbial ecology and compare it with wild-type S. mutans in vitro.

**Methods:**

Biofilm was evaluated for its polysaccharide content, scanning electron microscopy (SEM) imaging, acid-producing ability, and related lactic dehydrogenase (LDH), arginine deiminase (ADS), and urease enzymatic activity determination. Fluorescence in situ hybridization (FISH) and quantitative real-time polymerase chain reaction (qRT-PCR) were used to evaluate the S. mutans ratio within the biofilm. It was followed by 16S rRNA sequencing to define the oral microbial community.

**Results:**

Fluoride-resistant S. mutans produced increased polysaccharides in presence of NaF (P < 0.05). The enzymatic activities related to both acid and base generation were less affected by the fluoride. In presence of 275 ppm NaF, the pH in the fluoride-resistant strain sample was lower than the wild type. We observed that with the biofilm development and accumulative fluoride concentration, the fluoride-resistant strain had positive relationships with other bacteria within the oral microbial community, which enhanced its colonization and survival. Compared to the wild type, fluoride-resistant strain significantly increased the diversity and difference of oral microbial community at the initial stage of biofilm formation (4 and 24 h) and at a low fluoride environment (0 and 275 ppm NaF) (P < 0.05). Kyoto Encyclopedia of Genes and Genomes (KEGG) analysis revealed that fluoride-resistant strain enhanced the metabolic pathways and glucose transfer.

**Conclusions:**

Fluoride-resistant S. mutans affected the microecological balance of oral biofilm and its cariogenic properties in vitro, indicating its negative impact on fluoride's caries prevention effect.

## Introduction

Dental caries is among the most challenging diseases globally. Untreated caries in permanent teeth affected 2.4 billion people worldwide (Peres et al., 2019). Generally, the occurrence of caries is associated with the imbalance of acid and alkali production by the biofilms at the tooth surface (Pitts et al., 2017; Peres et al., 2019). A recently proposed caries ecology hypothesis suggests that the disease is related to the ecological homeostasis (Takahashi and Nyvad, 2011; Marsh et al., 2015). Ecological pressure leads to the imbalance of microorganisms or the abundance of pathogenic microorganisms such as *Streptococcus mutans*, resulting in the swift development of diseases (Takahashi and Nyvad, 2011). This theory prompted that controlling dental plaque is a critical point for the prevention and treatment of caries. As fluoride is widely used for preventing dental caries (Anusavice et al., 2005; Pitts et al., 2017), it enables the protection of the hard tissue of teeth by suppressing demineralization and accelerating remineralization (Ten Cate, 2004). Moreover, it influences the growth and metabolism of the bacteria (Oh et al., 2017). When fed into bacterial cytoplasm, fluoride reduces the activity of enolase and F-ATPases, decreases synthesis of intercellular polysaccharides and exopolysaccharides directly or indirectly, and weakens the acid-producing ability in the shape of hydrogen fluoride (Marquis et al., 2003; Liao et al., 2017). Nevertheless, due to its widespread use, fluoride also causes some problems. Dental fluorosis, fluorosis of bone, and fluoride resistance in bacteria commonly occur (Jha et al., 2011; Srivastava and Flora, 2020). For example, the appearance of a fluoride-resistant strain of *S. mutans* (FR) has been noted.

The FR strain can be isolated from xerostomia patients under high-dose fluoride treatment or laboratories (Streckfuss et al., 1980; Brown et al., 1983; Van Loveren et al., 1991a). *S. mutans* resistance to fluoride is no less than three times when compared with the fluoride-sensitive strain. Accordingly, studies on FR strains usually focus on their phenotype and genetic changes. Typical characters, such as fluoride resistance, adaptation, and stability, were identified, but cariogenic properties like acidogenicity and acidurity need special attention, due to their link with caries sensitivity (Zhu et al., 2012; Liao et al., 2015; Cai et al., 2017; Liao et al., 2017; Liao et al., 2018; Lee et al., 2021). As stable fluoride resistance was attributed to genetic changes (Van Loveren et al., 1991b; Liao et al., 2015; Liao et al., 2018; Lee et al., 2021), specific genes or their regulation were identified in causing fluoride resistance (Liao et al., 2016; Men et al., 2016; Murata and Hanada, 2016; Tang et al., 2019; Lu et al., 2020; Yu et al., 2020). Therefore, whether the rise of fluoride resistance would impact oral microbial ecology or not remains a question.

Recently, it was reported that fluoride resistance influenced the development of competitive dual-species biofilms comprising *S. mutans* and *Streptococcus sanguinis* (*S. sanguinis*) under *in vitro* fluoride treatment (Zhang K. et al., 2022). Fluoride resistance acquired an edge in competitive dual-species biofilm formation, resulting in a more robust biofilm formation, and increased cariogenic virulence. Nonetheless, the dual-species biofilm model is circumscribed for the lack of common representativeness. The oral microbial community is a typical multispecies flora colonized by more than 700 microbe species. Saliva biofilms are composed of a variety of bacteria, have the potential to restore the complexity of dental plaque, and reduce the enormous cost of animal models (Brown et al., 2019). This research used saliva-derived oral biofilm models that are easier to operate and repeat, especially in controlling variables when compared with animal models (Brown et al., 2019). In our study, we aim to comprehend how the FR strain makes a difference to the oral microbial communities in the presence of fluoride within biofilm development. Consequently, we compared saliva-derived biofilm’s cariogenic virulence caused by FR or wild-type *S. mutans* (WT) and characterized specific microbiome shifts regarding establishment and stability. Our findings provide systematic insight into the profound ecological influence of fluoride resistance.

## Materials and methods

### Microbial strains and culture conditions

Wild-type *S. mutans* UA159 (WT) and its fluoride-resistant strain (FR) were used in this study. The FR strain was generated *in vitro* as described previously (Zhu et al., 2012). Briefly, an overnight WT bacterial suspension was inoculated on brain heart infusion (BHI, Oxoid, Basingstoke, UK) agar plates containing varying concentrations of NaF (50 to 1000 ppm). Isolated colonies were picked and passaged on BHI agar without NaF for 50 generations. After passage, a clone growing on a BHI plate containing 1,000 ppm NaF was identified as an FR strain. Bacteria were cultured in BHI for multiplication in an atmosphere of 5% CO_2_ at 37°C.

### Saliva collection

Ethics was authorized by the School and Hospital of Stomatology, Wenzhou Medical University (WYKQ2020007). To eliminate the interference of other *S. mutans*, saliva was collected from a healthy donor, whose saliva did not contain any *S. mutans* (screened from 20 healthy volunteers) (Koopman et al., 2015; Uranga et al., 2021). Briefly, volunteers were informed to not brush their teeth for 24 h and prevent drink or food intake for 2 h, before the collection. Saliva was collected as previous study (Uranga et al., 2021). Total saliva DNA was extracted, and the absence of *S. mutans* was verified using quantitative real-time polymerase chain reaction (qRT-PCR, details are shown below). The primers are described in **Supplementary Table S1**. The collected saliva was mixed with 60% glycerol and stored at -80°C for further use (Huang et al., 2017).

Biofilms were cultured in 24-well plates containing glass disks in each well. Mixtures of saliva and *S. mutans* (WT or FR) were used for biofilm formation. The saliva sample was diluted 50-fold and mixed with an overnight culture of *S. mutans* (1.25 × 10^6^ cells per well) (Li et al., 2010). McBain medium with 0.2% sucrose was used to culture biofilms (McBain et al., 2005). Biofilms were incubated anaerobically (10% CO_2_, 10% H_2_, and 80% N_2_) for 4, 24, and 72 h with 0, 275, and 1,250 ppm NaF, and the medium was changed daily (Huang et al., 2017).

### Scanning electron microscopy

Biofilms were washed with PBS and fixed using 2.5% glutaraldehyde. Then, the biofilms were dehydrated using an ethanol gradient (50%, 60%, 70%, 80%, 90%, 95%, and absolute ethyl alcohol; 30 min incubation per concentration). Biofilms were dried, coated with gold-palladium, and imaged using a scanning electron microscope (SEM; Hitachi, Tokyo, Japan) at ×1,000 magnification (Zhang K. et al., 2022).

### Polysaccharide analysis

Quantitative analysis of water-insoluble polysaccharides was done by the anthrone method (Zhang K. et al., 2022). Briefly, the biofilms were collected, washed with PBS, resuspended in 0.4 M NaOH, and incubated for 30 min before centrifuging at 4,000×*g* for 10 min, and the supernatant was collected. The 100-μl supernatant was mixed with 300 μl anthrone solution (2 mg/ml, in concentrated sulfuric acid) and incubated at 95°C for 6 min in a water bath. The absorbance was monitored at 625 nm. Standard curves were prepared using the dextran standard.

Further, confocal laser scanning microscopy (CLSM) was performed to observe the polysaccharide production. Alexa Fluor 647 Dextran conjugate (Molecular Probes, Invitrogen Corp., Carlsbad, CA) was used to label α-glucan. After 4, 24, and 72 h of biofilm formation, biofilms were dyed with SYTO 9 (Molecular Probes, Invitrogen Corp., Carlsbad, CA, USA) and concanavalin A (Con A, (Molecular Probes, Invitrogen Corp., Carlsbad, CA, USA) to label microorganisms and α-D-glucopyranose polysaccharides separately (Chen et al., 2007; Adav et al., 2010). A CLSM (Nikon Corporation, Tokyo, Japan) was used to obtain the image, with excitation/emission spectrums of 650/668 nm for Alexa Fluor 647, 555/580 nm for conA, and 480/500 nm for SYTO 9 (Zhang L. et al., 2022).

### Acid production analysis

The acid-producing ability of biofilms was studied by measuring the pH and lactic acid content. After 4, 24, and 72 h incubation of biofilms, the supernatant pH was measured using a pH meter (Mettler Toledo Instruments Co. Ltd., Shanghai, China).

After biofilm formation, the biofilms were washed with Cysteine Peptone Water (CPW) and transferred to a new 24-well plate. Buffered Peptone Water (BPW, 1 ml) containing 0.2% (v/v) sucrose was added to each well and incubated at 37°C for 3 h to produce acid. The lactic acid content was measured by an enzymatic method (Zhang L. et al., 2022).

### Enzyme activity determination

Protein content was used to standardize arginine deiminase (ADS) and urease activity and defined as µmol/min/mg protein (Zheng et al., 2017). In brief, cells from saliva-derived biofilms were added into a mixture with 50 mM arginine hydrochloride (Sigma-Aldrich Canada, Oakville, Ontario, Canada) and 0.5 mM Tris-maleate buffer (pH 6.0) and then incubated together for 120 min at 37°C to allow ammonia generation. The ammonia production was monitored using Nessler’s reagent (Sigma-Aldrich) based on a standard generated with ammonium sulfate. Simultaneously, protein content was measured using Bradford’s assay and bovine serum albumin was used as standard. Lactic dehydrogenase (LDH) activity was determined using an LDH Activity Assay Kit (Sigma-Aldrich), as per the manufacturer’s guidelines (Zheng et al., 2017). Further, a deviation between the fluoride group (275 and 1250 ppm NaF) and the control group (0 ppm NaF) for the FR strain and WT was calculated separately. Results were shown as the absolute value of the deviation of enzyme activities compared with corresponding 0 ppm (ΔLDH, ΔADS, and ΔUrease).

### Fluorescence *in situ* hybridization

Fluorescence *in situ* hybridization (FISH) was used to monitor *S. mutans* within biofilms (Zhang K. et al., 2022). Briefly, biofilms were fixed with 4% paraformaldehyde, treated with lysozyme, and dehydrated using an ethanol gradient (50%, 80%, and 96%). *S. mutans* and whole bacteria were dyed using specific probes (**Supplementary Table S2**). Biofilm images were obtained and analyzed using a CLSM (Nikon A1, Nikon Corporation, Japan) equipped with an oil immersion lens at ×60.

### qRT-PCR

The quantitative ratio of *S. mutans* within saliva-derived biofilms was determined by qRT-PCR (Zhang K. et al., 2022). Genomic DNA was extracted from biofilms using a QIAamp DNA Mini Kit (QIAamp, Germany), as per the manufacturer’s instructions. For qRT-PCR, a 20-μl reaction mixture was used (primers and probes are listed in **Supplementary Table S1**). The assay reaction was run in a StepOnePlus Real-Time PCR System (Applied Biosystems, Waltham, MA) as follows: 95°C for 30 s, 40 cycles of 95°C for 10 s, and 58°C for 30 s (Yoshida et al., 2003).

### 16S rRNA gene sequencing and analyses

Sequencing was used to detect the FR’s ecological impression. After biofilm formation, the biofilm was collected by centrifugation followed by its transportation to Shanghai Majorbio Bio-Pharm Technology Co., Ltd. (Shanghai, China). DNA was extracted, amplified, and sequenced based on standard procedures. In brief, DNA was extracted utilizing FastDNA^®^ Spin Kit (MP Biomedicals, USA). Sense primer (5′-CCTAYGGGRBGCASCAG-3′) and anti-sense primer (5′-GGACTACHVGGGTWTCTAAT-3′) were used for PCR amplification (Liu et al., 2016). Sequencing procedures were run with an Illumina NovaSeq PE250 platform (Illumina, San Diego, USA). The analyses of sequence data were performed using fastp version 0.19.6, FLASH version 1.2.7, PICRUSt2 (Phylogenetic Investigation of Communities by Reconstruction of Unobserved States), and UPARSE 7.1, involving raw data quality control, taxonomic annotation based on the NCBI database (Wang et al., 2007; Magoc and Salzberg, 2011; Edgar, 2013; Chen et al., 2018; Douglas et al., 2020). Bioinformatics and statistical analyses were as follows. The relative abundance difference of microbic taxa was examined at species levels by the Kruskal–Wallis test. The α-diversity between groups was performed using the Shannon index (Schloss et al., 2009). Principal coordinate analyses (PCoAs) were generated to calculate the community difference between groups, along with two non-parametric analyses, involving analysis of similarities (ANOSIM) and non-parametric multivariate analysis of variance (Adonis) using distance matrices. The co-occurrence network at the species level was analyzed using the Spearman correlation matrix (ρ > 0.6 and *P* < 0.01) (Barberán et al., 2012). Microbial functions (pathway and enzyme) were predicted by phylogenetic investigation of communities to rebuild the unobserved states based on the 16S rRNA gene sequence data. The rarefaction curves of samples are shown in **Supplementary Figure S1**.

### Statistical analysis

All the assays were repeated three times independently. The statistical analysis was done using Statistical Package for Social Sciences (SPSS 16.0, SPSS Inc., Chicago, IL, USA). ANOVA and Scheffé *post-hoc* comparison were applied for comparison. A statistically significant difference was observed with *P* < 0.05.

## Results

### FR-involved biofilms produced more polysaccharides under fluoride and affected biofilm architecture differently

**Figure 1:**
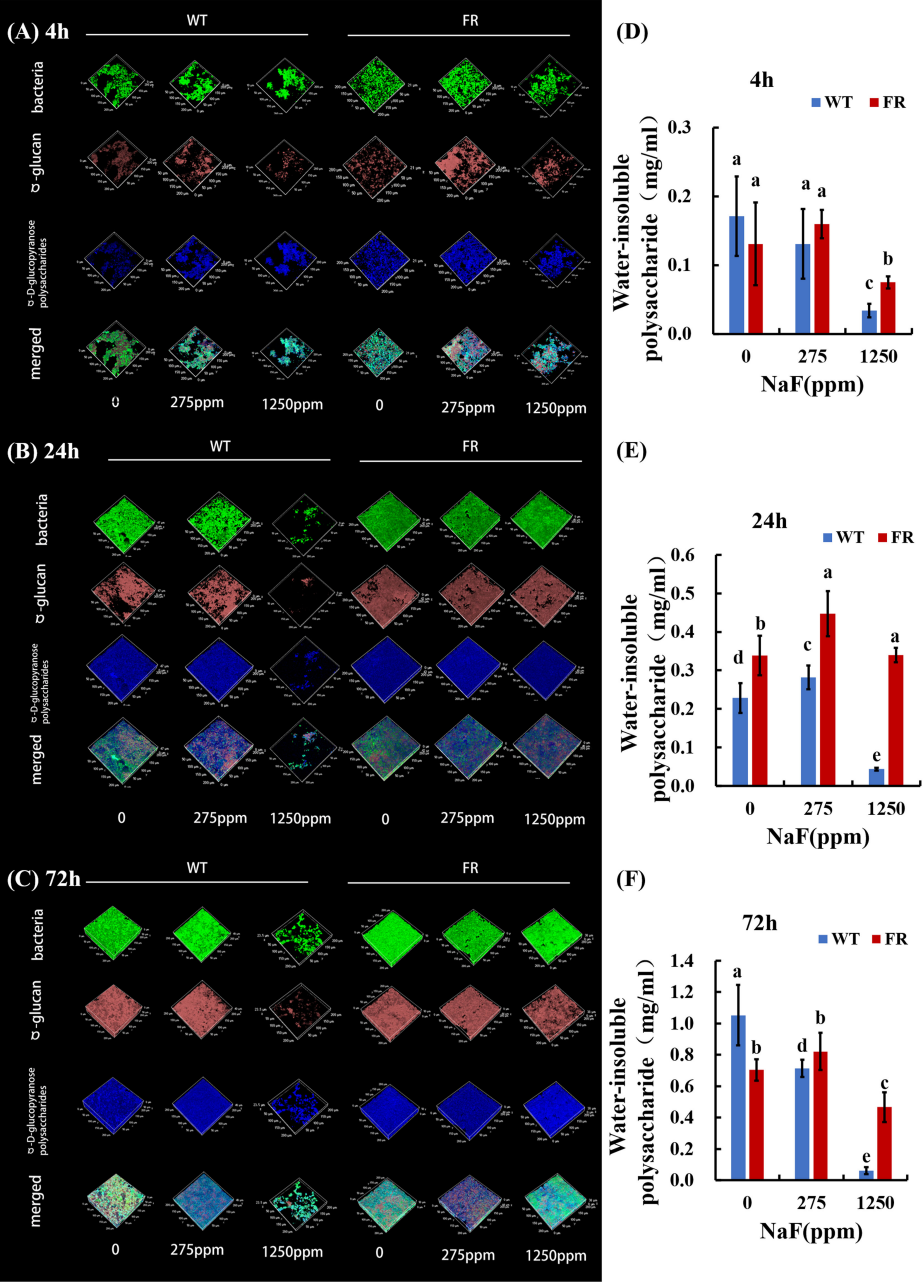
Polysaccharide analysis of biofilms. **(A-C)** Bacterial and polysaccharide staining of biofilms. Bacteria were labeled with green, dextran was stained red, and α-polysaccharides were dyed blue. **(D-F)** Water-insoluble polysaccharide of biofilms revealed by anthrone mensuration. Data are presented as mean ± standard deviation, and different letters demonstrate a significant difference between groups (P < 0.05).

**Figure 2:**
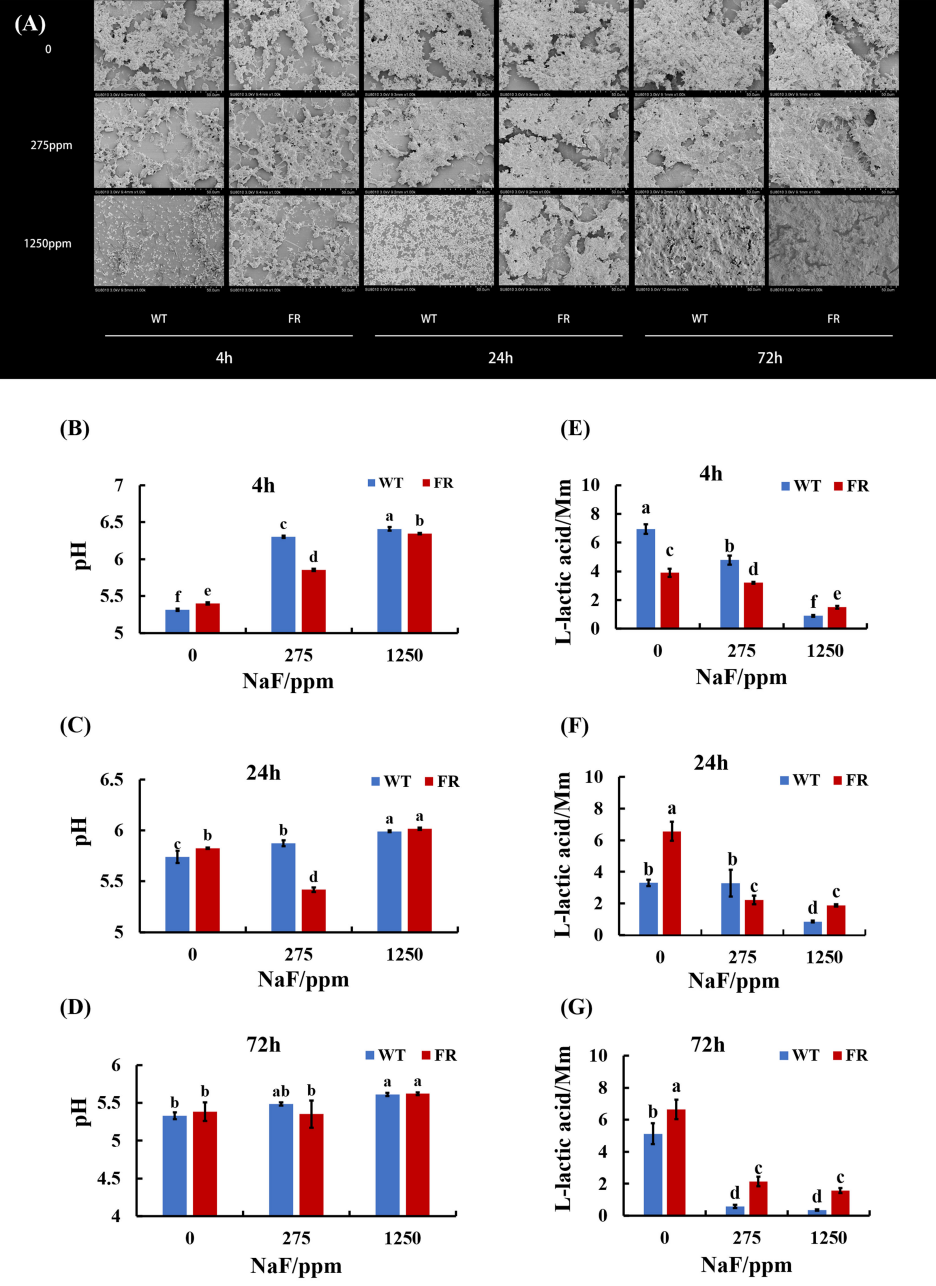
Biofilm structure and acid production. **(A)** SEM images of biofilms, scale bar 50 μm. **(B-D)** Supernatant pH of biofilms. **(E-G)** Lactic acid generation of biofilms. Data are presented as mean ± standard deviation, and different letters demonstrate a significant difference between groups (P < 0.05).

An obvious alteration of EPS information and biofilm construction can be found in CLSM and SEM images. CLSM images confirmed that in EPS staining (**Figures 1A-C**), biofilms with FR had more thickness and EPS than the WT ones, at different time points. The difference was particularly pronounced at a high NaF concentration (1,250 ppm). As shown in **Figures 1D-F**, under fluoride exposure, water-insoluble exopolysaccharide of FR-related biofilms increased more visibly than WT, at each of the three time points (*P* < 0.05), except for the 4-h biofilm at 275 ppm, among which there was no significant difference though. The SEM images (**Figure 2A**) showed a somewhat similar tendency. Collectively, the existence of FR led to biochemical and structural alternation of biofilms, resulting in more robust biofilms with more biomass under fluoride when compared with the ones with WT.

### FR-involved biofilms had lower pH at 275 ppm NaF and showed stronger acidogenicity after high fluoride treatment

FR groups had lower supernatant pH than WT groups at 275 ppm NaF (**Figures 2B-D**). The accessorial supernatant pH with the increase in fluoride implied a repressive effect by fluoride on the biofilm acid output. However, we observed that in lactic acid generation, the effect of fluorine was different. For each age of saliva-derived biofilms (4, 24, and 72 h), the lactic acid production increased with fluoride resistance to 1,250 ppm compared with its counterpart WT (**Figures 2E-G**; *P* < 0.05). It showed a similar tendency at 0 (24 h biofilms) and 275 ppm (72 h-biofilms) (**Figures 2F, G**; *P* < 0.05). Hence, the data showed that the acidogenicity advantage of saliva-derived biofilms with FR could appear after high fluoride treatment.

### FR-involved biofilms were more likely to maintain the primary acid–base metabolism of oral microorganisms facing fluoride stress

**Figure 3:**
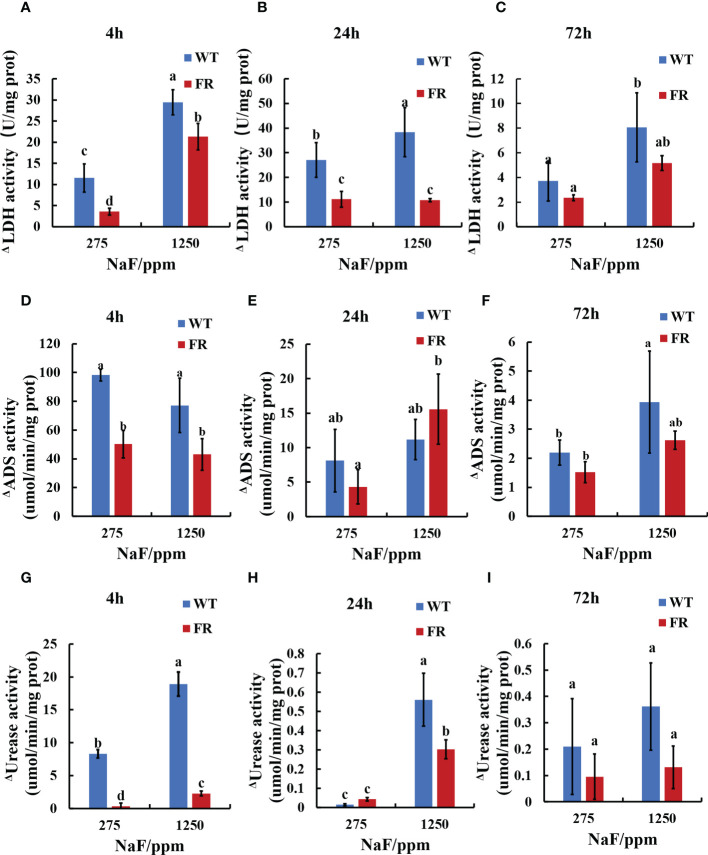
Enzyme activity in biofilms. **(A-C)** LDH activity. **(D-F)** ADS activity. **(G-I)** Urease activity. Δ represents shape of the absolute value of deviation of enzyme activities when compared with corresponding 0 ppm. Data are presented as mean ± standard deviation, and different letters demonstrate a significant difference between groups (P < 0.05).

Enzyme activity related to acid–base metabolism was detected for its close connection with cariogenicity. For enzymatic activity, lactic dehydrogenase (LDH, acidogenic enzyme), arginine deiminase (ADS, alkali-producing enzyme), and urease (alkali-producing enzyme) activities were investigated and deviation was calculated between the fluoride group (275 and 1250 ppm NaF) and control group (0 ppm NaF) for the FR strain and WT separately (**Figure 3**). The deviations of FR groups were lower than WT groups in general (**Figure 3**), except ΔADS activity for 24-h biofilms at 1,250 ppm NaF and ΔUrease activity for 24-h biofilms at 275 ppm NaF, which showed a different trend but with no significant difference (**Figures 3E, H**, *P*>0.05). Although deviations of FR groups including ΔADS activity, ΔADS activity, and ΔUrease activity in all the remaining groups were lower than those of WT ones, no significant differences were observed between FR and WT groups for ΔLDH activity (72-h biofilms), ΔADS activity (24-h biofilms at 275 ppm NaF and 72-h biofilms), and ΔUrease activity (72-h biofilms) (**Figures 3C, E, F, I**, *P*>0.05). A significant difference can be found in ΔLDH activity (4-h biofilms and 24-h biofilms), ΔADS activity (4-h biofilms at 275 and 1,250 ppm NaF), and ΔUrease activity (4-h biofilms at 275 ppm and 1,250 ppm NaF together with 24-h biofilms at 1,250 ppm) (**Figures 3A, B, D, G, H**, *P* < 0.05). These findings indicate that under fluoride exposure, FR groups held to previous acid–base metabolism rather than building a new one for oral microorganisms compared with the WT groups.

### FR acquired a competitive advantage and changed microbial composition

**Figure 4:**
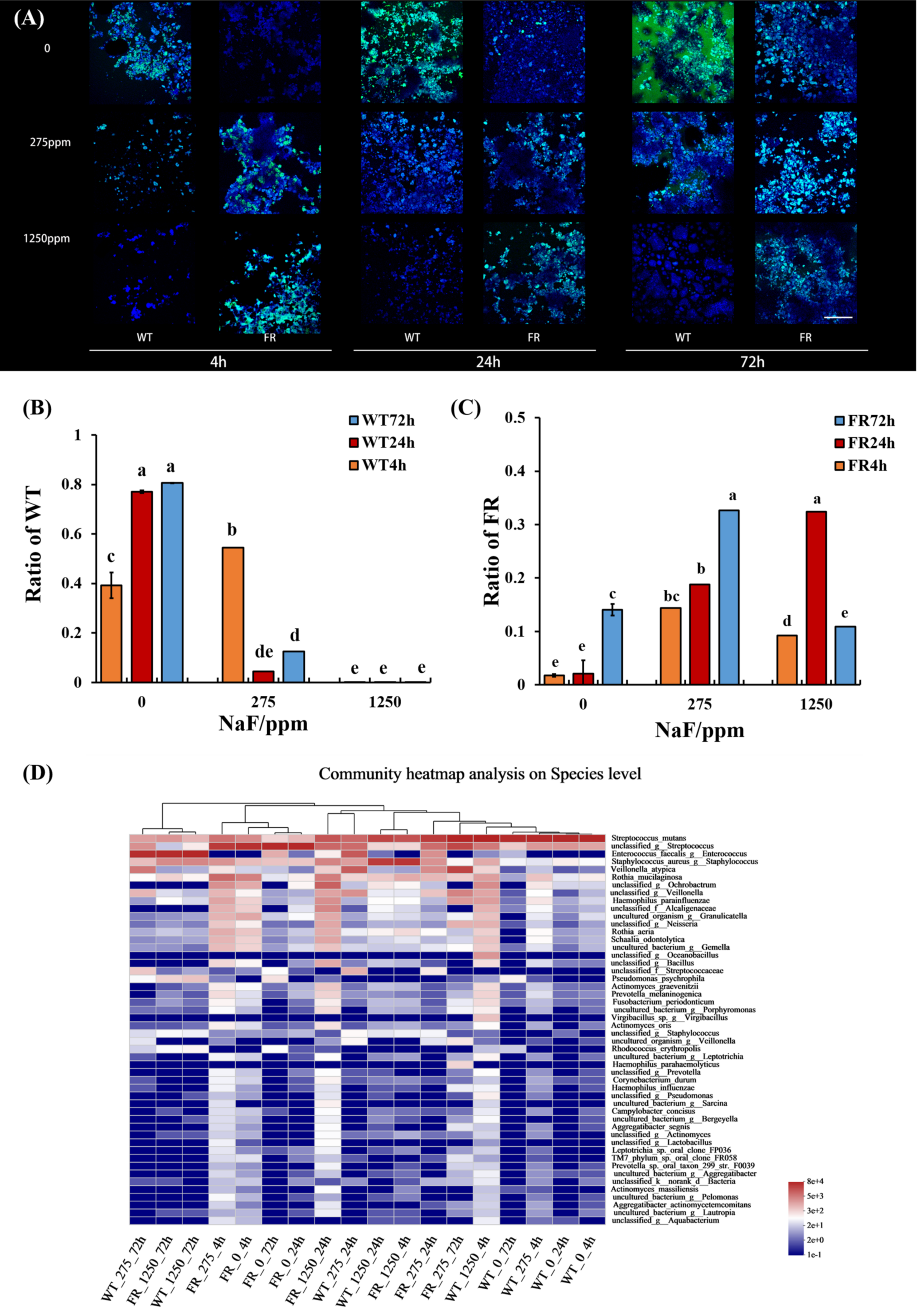
*S. mutans* ratio in biofilm and relative abundance of the 50 most predominant oral bacteria at the species level. **(A)** *S. mutans* within biofilms revealed by FISH; *S. mutans* was stained green, and all bacteria were stained blue, the scale bar 50 μm. **(B)** WT ratio revealed by qRT-PCR. **(C)** FR ratio revealed by qRT-PCR. **(D)** Relative abundance of the 50 most predominant oral bacteria at the species level. Data are presented as mean ± standard deviation, and different letters demonstrate a significant difference between groups (P < 0.05).

To better delineate the influence of FR on the oral microbial community during the development of saliva-derived biofilm, we investigated the ratio of WT and FR by qRT-PCR and FISH and the percentages of community abundance on a species level by 16S rRNA sequencing. Both FISH and qRT-PCR appeared at a similar ratio (**Figures 4A-C**, *P* < 0.05). In general, with increasing NaF concentrations (except group at 4 h and 275 ppm NaF), the WT proportion reduced in the biofilms and the FR proportion increased (**Figures 4A-C**, *P* < 0.05). A similar trend can be found in 16S rRNA sequencing results (**Figure 4D**). With time and the increasing concentration of NaF, FR exhibited a competitive advantage and its advantage was most remarkable in 24-h biofilms at 275 ppm (**Figure 4D**, *P* < 0.05). Surprisingly, despite the higher percentages of FR colonies than WT, the FR strains did not reach overwhelming superiority in saliva-derived biofilms, under a high-fluorine environment (1,250 ppm NaF) in contrast to a low-fluoride environment (275 ppm NaF) (**Figure 4D**, *P* < 0.05), except in 24-h biofilms. Meanwhile, the percentage of WT gradually decreased with the increase in time and concentration of NaF (**Figure 4D**, *P* < 0.05). The composition of microbial communities incubated with NaF was notably different from that with WT (**Figure 4D**). Given these, we documented that fluoride resistance obtained a competitive advantage under NaF and affected microbial composition.

### FR made the microbe within biofilms more diverse and distinct in the early biofilm establishment phase at low fluoride

**Figure 5:**
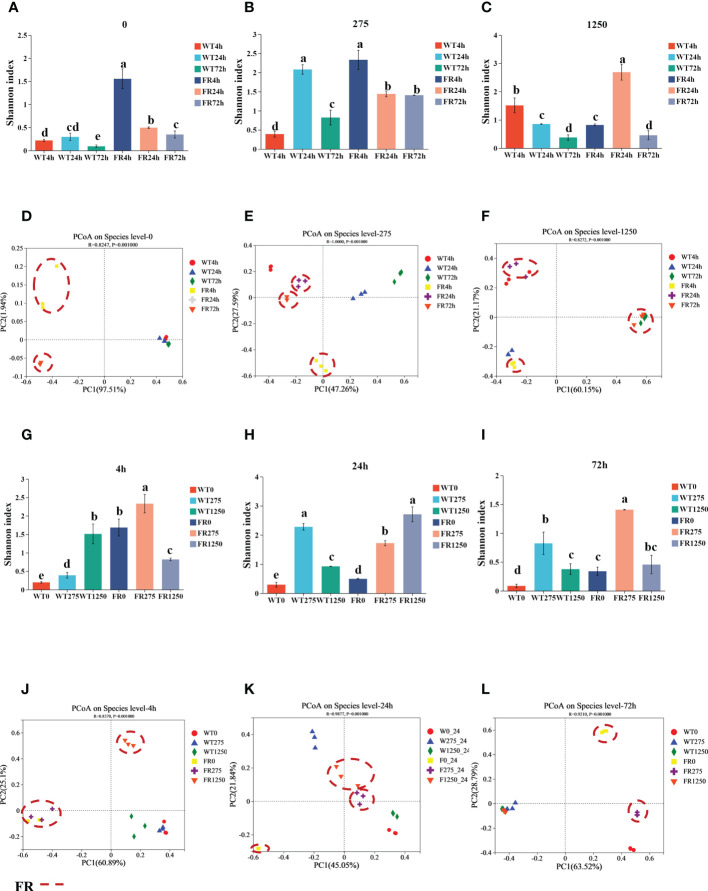
Shannon indices and PCoA of saliva-derived oral biofilms in the presence of WT/FR. **(A-C)** The Shannon indices separately at different incubating times of each concentration of NaF (0, 275, 1,250 ppm), standing at 4, 24, and 72 h. **(G-I)** The Shannon indices respectively at distinctive concentrations of NaF of each timepoint (4, 24, 72 h), representing 0, 275, and 1,250 ppm. **(D-F)** PCoA separately at different incubating times of each concentration of NaF (0, 275, 1,250 ppm), standing at 4, 24, and 72 h. **(J-L)** PCoA respectively at distinctive concentrations of NaF of each timepoint (4, 24, 72 h), representing 0, 275, and 1250 ppm. Data are presented as mean ± standard deviation, and different letters demonstrate a significant difference between groups (P < 0.05).

We examined the impression of FR on microbial ecology in saliva-derived biofilms by the method of 16S rRNA sequencing (values of Shannon index and PCoA of all groups are shown in **Supplementary Figure S2**). As shown in **Figure 5B**, fluoride resistance played an important role on the microbial diversity in a low-fluoride environment (275 ppm), based on Shannon indices (*P* < 0.05). The case was similar at 0 ppm; at 275 ppm, the Shannon index of the group was higher than the 0-ppm group (**Figures 5A, B**; *P* < 0.05). PCoA showed that the oral microbial communities in the presence of FR were distinct from the WT; nevertheless, the difference was no longer so apparent with time and increasing concentration of NaF, which is also indicated by the two dissimilarity tests including Adonis and ANOSIM (**Figures 5D-F, J-L**; **Table 1**; *P* < 0.05), whereas there was no significant difference between the figures of FR and WT at 1,250 ppm overall (**Supplementary Figure S3F**; *P* > 0.05). The community diversity of the 4- and 24-h biofilms altered with FR, and the values of the Shannon index were close in these two groups (**Figures 5G, H**; P < 0.05). With increasing incubation time (72 h), the values of the Shannon index became more similar between the WT and FR groups (**Supplementary Figure S3I**, *P* > 0.05). By contrast, no significant differences were observed between the WT and FR groups’ Shannon indices for 275 ppm and 24 h (**Supplementary Figures S3B, H**, *P* > 0.05). There was no significant difference in the observations for FR and WT groups at 1,250 ppm and 72 h (**Supplementary Figures S3C, I**; *P* > 0.05), although its counterparts at different timepoints in the 1,250-ppm group and different concentrations of NaF in the 72-h group can distinguish respectively (**Figure 5C**; *P* < 0.05). These data show that during the early establishment of oral microbes (4 h, 24 h), FR played a role in adding diversity and distinction of communities, which was also present at low-fluoride concentrations (275 ppm). Nonetheless, the dissimilarities in diversity and distinction were gradually not so obvious with the increase in biofilm age (72 h) and concentration of NaF (1250 ppm).

**Table 1:** Dissimilarity tests (Adonis and ANOSIM) for the biofilm microbiota.

	ANOSIM		Adonis	
	*R*	*P*	*R^2^*	*P*
Overall	0.9024	0.001	0.9577	0.001
Saliva + FR *vs*. saliva + WT-0	0.8247	0.001	0.9954	0.001
Saliva + FR *vs*. saliva + WT-275	1	0.001	0.9738	0.001
Saliva + FR *vs*. saliva + WT-1250	0.8272	0.001	0.9056	0.001
Saliva + FR *vs*. saliva + WT-4h	0.837	0.001	0.9025	0.001
Saliva + FR *vs*. saliva + WT-24h	0.9877	0.001	0.9627	0.001
Saliva + FR *vs*. saliva + WT-72h	0.8272	0.001	0.9897	0.001

### FR obtained more help for colonization in microbial communities with biofilm development

**Figure 6:**
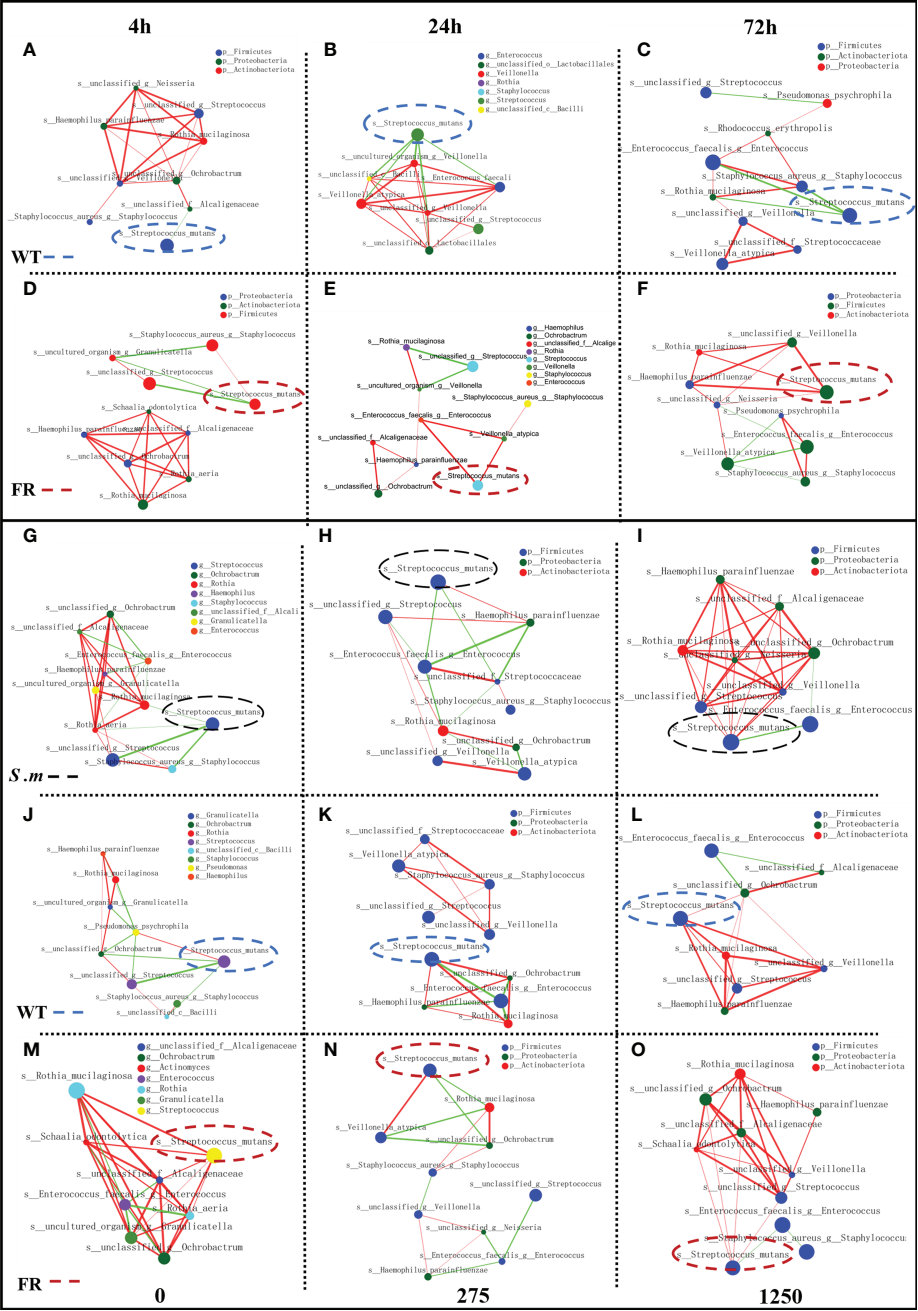
Network inferences of microbial relationships in oral biofilms with the presence of WT/FR. Each node represents an OTU, and each edge represents a significant pairwise association. Green lines represent negative relationships, and red lines represent positive relationships. **(A-C)** The network inferences of microbial relationships separately at different incubating times among biofilms with WT, standing at 4, 24, and 72 h. **(D-F)** The network inferences of microbial relationships separately at different incubating times among biofilms with FR, standing at 4, 24, and 72 h. **(G-I)** The network inferences of microbial relationships respectively at distinctive concentrations of NaF in biofilms with the presence of *S. mutans*, representing 0, 275, and 1,250 ppm. **(J-L)** The network inferences of microbial relationships respectively at distinctive concentrations of NaF in biofilms with the presence of WT, representing 0, 275, and 1,250 ppm. **(M-O)** The network inferences of microbial relationships respectively at distinctive concentrations of NaF in biofilms with the presence of FR, representing 0, 275, and 1,250 ppm.

Co-occurrence ecological networks concerning the top 10 abundant species-level taxa were constructed to further comprehend how saliva-derived biofilms assemble as incubation time and concentration of NaF increased and whether colonization of the FR strain impacted the oral microbial community network topology. With time, the positive relationship between the FR strain and other species increased gradually and the negative was reduced (**Figures 6D-F**). The negative relationship in the WT group was most strengthened for the 24-h biofilm (**Figures 6A-C**). However, the situation changed when the concentration of NaF was considered. The negative links between *S. mutans* and other species decreased following the gradual increase of NaF (0, 275, and 1,250 ppm), whereas the positive one was the opposite (**Figures 6G-I**). The tendency of WT groups was similar to the one of the whole (**Figures 6J-L**). In contrast, for the FR groups, the relationship was slightly reduced (**Figures 6M-O**). Thus, it was documented that with biofilm development, the FR strain was easier to colonize in the communities and it can build a more positive relationship with other species, which increased the risk of caries. However, this situation may not be suitable under NaF.

### FR might influence metabolism-related pathways and enhance glucosyl transfer within biofilms

**Figure 7:**
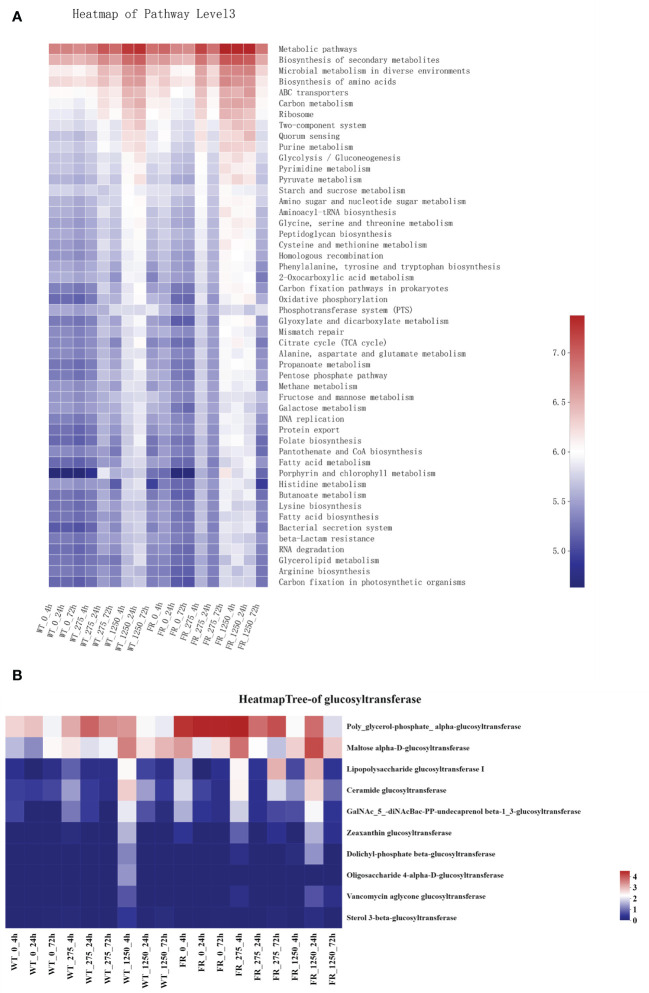
Prediction of Kyoto Encyclopedia of Genes and Genomes pathways and major glucosyltransferase of biofilms. **(A)** Prediction of KEGG pathways. The color gradient of the color block is used to display the changes in the abundance of different functions in the groups, and the legend indicates the value represented by the color gradient. **(B)** Major glucosyltransferase of biofilm in the presence of WT/FR. The color gradient of the color block is used to display the changes in the abundance of different functions in the groups, and the legend indicates the value represented by the color gradient.

Metabolism and biosynthesis related, ABC transporters involved, two-component system and quorum sensing associated as per Predicted Kyoto Encyclopedia of Genes and Genomes (KEGG) were upregulated in saliva-derived biofilms cultured with FR when compared with WT groups (**Figure 7A**). Similarly, most KEGG enzymes related to glucosyl transfer also enhanced within biofilms with FR when compared with WT groups (**Figure 7B**).

## Discussion

Complex microbial communities inhabit the oral cavity, where the abundance and composition of microbial species shift resulting in ecological imbalance leading to polymicrobial diseases such as dental caries (Grice et al., 2008; Dewhirst et al., 2010; Fujimura et al., 2010; Hajishengallis, 2015; David et al., 2014; Levy et al., 2017). This study for the first time reveals the influence of FR on oral microbial communities under fluoride exposure *in vitro*. Our data indicated that the presence of FR impacted polysaccharide generation, acidogenicity, acid–base metabolism, enzyme activity, and microbial composition *in vitro*.

EPS is vital for the establishment of oral biofilms and the development of dental caries (Flemming and Wingender, 2010; Koo et al., 2013). Microbial communities of biofilm are wrapped in an extracellular matrix composed of polymers like EPS, which serves as 3D architecture, diffusion barrier, and cariogenic combining site (Flemming and Wingender, 2010; Xiao et al., 2012; Koo et al., 2013). EPS accelerates adherence of *S. mutans* on the surface of teeth by salivary glucosyltransferase (Vacca-Smith and Bowen, 1998; Bowen and Koo, 2011). One research reported that the cell surface polysaccharide biosynthesis-related genes in *Acidithiobacillus ferrooxidans* (*A. ferrooxidans*) can be expressed more under fluoride (Ma et al., 2016). Additionally, it has been observed that *malQ* genes in the *S. mutans* FR strain were upregulated, which is annotated as 4-alpha-glucanotransferase (Lee et al., 2021). Our findings illustrated that compared with the WT, more EPS was produced in FR groups under fluoride exposure (**Figures 1** and **2A**). In addition, the expression of glucosyltransferase was upregulated in saliva-derived biofilms with FR under fluoride exposure (**Figure 7B**). Given this, we guessed that owing to better adaptability under fluoride, FR enhanced the activity of glucosyltransferase, produced more EPS, and further contributed to the cariogenicity of an oral biofilm under NaF.

The accumulation of acid in an oral environment causes the demineralization of teeth directly and alters oral microbial flora indirectly (Takahashi and Nyvad, 2011; Pitts et al., 2017). *S. mutans* was identified to play an important role in caries not only for its EPS-matrix generation ability and aciduricity but also for its cariogenicity. FR biofilms had lower pH at 275 ppm NaF at three time points, which were consistent with a previous study in which FR-related dual-species biofilms had lower supernatant pH at 275 ppm NaF whereas less difference was found at 1,250 ppm NaF (Zhang K. et al., 2022). Under low fluoride, FR may be responsible for enhanced cariogenicity depending on its better survivability and acid production under NaF, whereas the inhibitory effect of NaF on acid generation may play a dominant role at high NaF concentrations, hence less difference between FR and WT groups in addition to higher pH when compared with 275 ppm NaF. Yet, these conjectures need to be studied. Lactic acid results showed higher acidogenicity at high fluoride (1,250 ppm NaF) treatment, whose additional acid production may increase the demineralization of teeth (**Figures 2E-G**) that may result from more total biomass and higher *S. mutans* ratio in FR groups at this concentration.

LDH, an oxidoreductase isoenzyme, acts as a catalyst for reaction of lactic acid and pyruvate. ADS can turn arginine to ammonia, and urease can hydrolyze urea to ammonia, to counteract the effects of biofilm acidification caused by bacterial glycolysis, which are the main alkaline-producing enzymes in oral bacteria (Colby and Russell, 1997). Interestingly, the data exhibited that changes in LDH, ADS, and urease activities in oral biofilms cultured with the FR strain were lower than WT or unchanged (**Figure 3**). Extremophiles such as fluoride-resistant strain have survival strategies to adjust themselves to suit an extreme environment (Raddadi et al., 2015). It has been reported that biofilm’s maturation can affect the anticaries efficacy of fluoride including LDH activity (Ayoub et al., 2022). We speculate that in FR groups, the priority is to restore primary metabolic disequilibrium instead of establishing a new one under conditions of fluoride stress, so that it can enhance fluoride tolerance.

We find that FR strains would obtain overwhelmingly competitive advantage in oral microbial communities with biofilm development and fluoride, although it was less than WT at the beginning (**Figure 4**). It was apparent that the FR strain gradually showed its fluoride endurance in the colonizing process in the oral environment, especially with fluoride. A former study also reported that FR can acquire competitive advantage in dual-species biofilms in biofilm formation, which is consistent with our results. We see that with the application of oral care products with fluoride, FR can also result in some adverse impact. Its prosperous growth might lead to dental caries as one of major pathogenic bacteria.

In recent years, research has observed that there is a close connection between modern diseases and dysbiosis of oral microbiota (Hajishengallis, 2015; Levy et al., 2017). We documented that FR enables oral microbial communities to be more diverse and rich in the early formation phase (4 and 24 h) of biofilm and at low fluoride (275 ppm), which may lead to more severe dysbiosis than WT (**Figures 5B, G, H**) (Li et al., 2004). The reason may be the better fluoride tolerance of the FR strain during fluoride stress, and at 275 ppm, the diversity of communities was enhanced compared with those with no fluoride (**Figures 5A, B**). One research has reported that salivary microbiota was resistant to a microbial shift (Belstrøm et al., 2018). In other words, it is likely to restore to their original with time. As the current study showed, with the development of biofilms, oral microbial communities gradually established, and till 72 h, it may restore to their original. Thus, the 72-h saliva-derived biofilms cocultured with FR became similar to WT in diversity (**Figure 5I**). Fluoride toxicity is significant at high concentrations (Li et al., 2013). We speculate that during fluoride stress, limited species of bacteria could survive; hence, there was no significant diversity between two groups at 1,250 ppm NaF (**Figure 5C**). Likewise, it can also explain why with the increasing age of biofilm and NaF concentration, the distinction of oral microbial communities between the FR and WT groups was not so obvious as observed in the early colonization phase and under low NaF (**Figures 5D-F, J-L**).

An interesting phenomenon was revealed by co-occurring ecological networks with the increase in the biofilm’s maturation; the positive relationship between FR and other species was reinforced, and the negative one was weakened. It can exhibit the adaptation of FR faced with fluoride, especially compared with the case of WT (**Figure 6**). Adaptation is the most effective solution for environmental changes (Bowden, 1990), and it has been demonstrated that fluoride has the potential to provide some bacteria with the ecological benefits and some bacteria could adapt to fluoride (Hamilton and Bowden, 1982; Bowden, 1990). Thus, our results may also indicate the willingness of other species in the oral biofilm to adapt to the fluoride stress. We infer that the total environment can be considered favorable for the FR colony, due to the positive relationship between the FR strain and other species (Costello et al., 2009). With time, the relationship strengthened followed by the increased risk of caries. The abnormality in the case of NaF may be explained by the fluoride toxicity dimension (Li et al., 2013). The increase of NaF exhibits a toxic effect on the microorganisms; hence, the density and diversity of friendly species may decrease sharply. This result is consistent with other research that showed that the presence of FR in the oral environment would not necessarily reduce the anti-caries effect of fluoride.

Expressions of metabolism- and biosynthesis-related KEGG pathways were enhanced in the oral biofilms cultured with FR (**Figure 7A**). It has been reported that gene expression related to fluoride resistance upregulated the energy metabolism and protein synthesis (Ma et al., 2016). Metabolism-related pathways play an important role in physiological balance and protein biosynthesis and are crucial for cell growth under fluoride-like carbon metabolism (Xu et al., 2014; Joshua, 2019; Judge and Dodd, 2020). In addition, ABC transporter-related KEGG pathways were also heightened in FR groups, which was consistent with a previous study in which ABC transporters and permeases were enhanced in an FR strain (Lee et al., 2021). They hypothesized that upregulation of ABC transporters and permeases was to export fluoride ions (Lee et al., 2021). Furthermore, two-component system-related (involved in sensing and responding to environmental change) and quorum sensing-related (responsible for microbial communication and regulating associated physiological characteristics such as biofilm formation) KEGG pathways were upregulated in FR groups under NaF. Combining these results and upregulation of EPS-related genes as mentioned above, we speculated that the FR group can be superior to WT groups in coping with NaF stress in the following ways. FR groups showed more activity in sensing and communication within biofilms, followed by regulation of biofilms to adapt to stress by forming more robust biofilms and provide stronger protection simultaneously, pumping out fluoride ions and providing necessary energy and substances. However, these conjectures required further investigation. The inadequacy of the current investigation was the shortage of animal text to identify findings. In addition, using drugs such as arginine to regulate oral biofilm ecologically might be a noteworthy attempt to decrease adverse impacts resulting from FR.

Overall, the emergence of FR would affect the microecological balance of oral biofilms and their cariogenic properties *in vitro*. Their appearance may also affect the caries prevention effect of fluoride.

## Data availability statement

The datasets of 16S rRNA gene sequencing presented in this study can be found in online repositories (https://www.ncbi.nlm.nih.gov/bioproject/PRJNA904954). Further inquiries can be directed to the corresponding authors.

## Ethics statement

The studies involving human participants were reviewed and approved by School and Hospital of Stomatology, Wenzhou Medical University. The patients/participants provided their written informed consent to participate in this study.

## Author contributions

YP and YSu designed this project. YSh, FY and LQ conducted experiments and acquired the data. MG, PX, XL, MW and XH analyzed and interpreted the data. YP and YSu polished the language. YSh wrote the main manuscript text. YP and YSu acquired funding. All authors contributed to the article and approved the submitted version.
