# Supplementary material for: Ecological influence by colonization of fluoride-resistant Streptococcus mutans in oral biofilm
Source: Front Cell Infect Microbiol. 2023 Jan 9;12:1106392. doi: 10.3389/fcimb.2022.1106392 (PMC9868560; doi:10.3389/fcimb.2022.1106392)
Supplement: Supplementary file 1 [file DataSheet_1.docx]

Supplementary Material

#
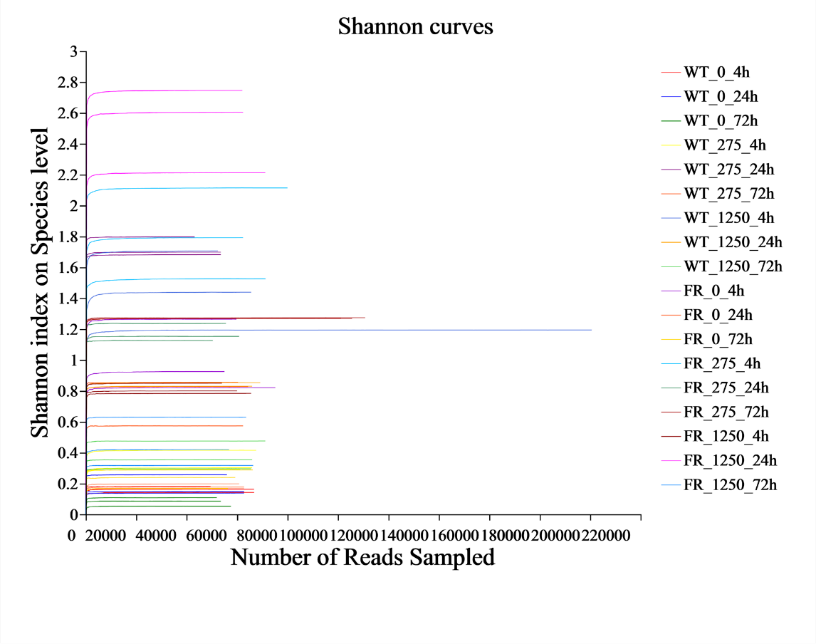
Supplementary Figure 1

**Supplementary Figure S1.** The rarefaction curves which showed clear asymptotes indicating that a near- complete sampling of the community.


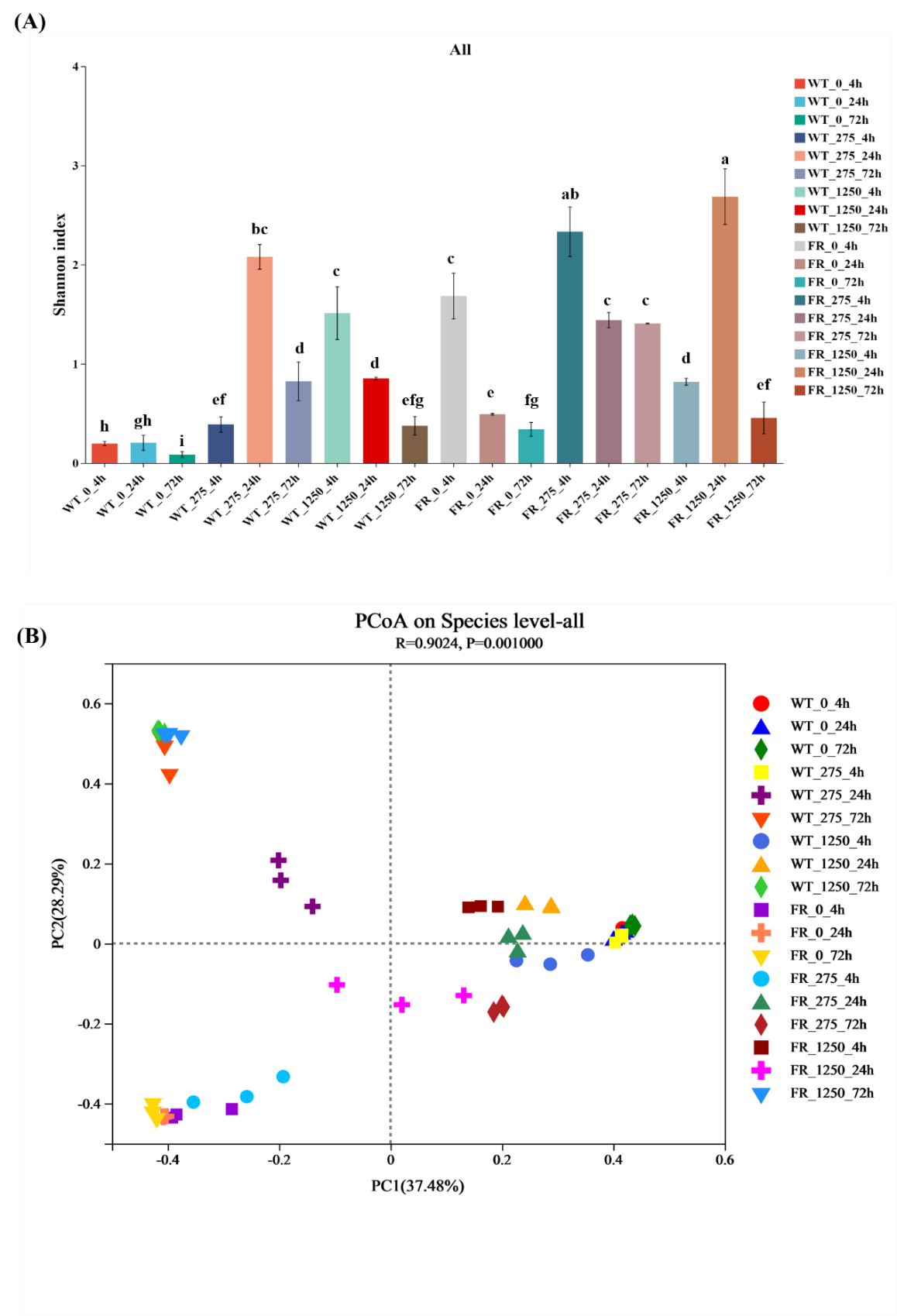


**Supplementary Figure S2.** Values of Shannon index (A) and PCoA analysis (B) of all groups. Data are presented as mean ± standard deviation and different letters demonstate a significant difference between groups (P ＜ 0.05).


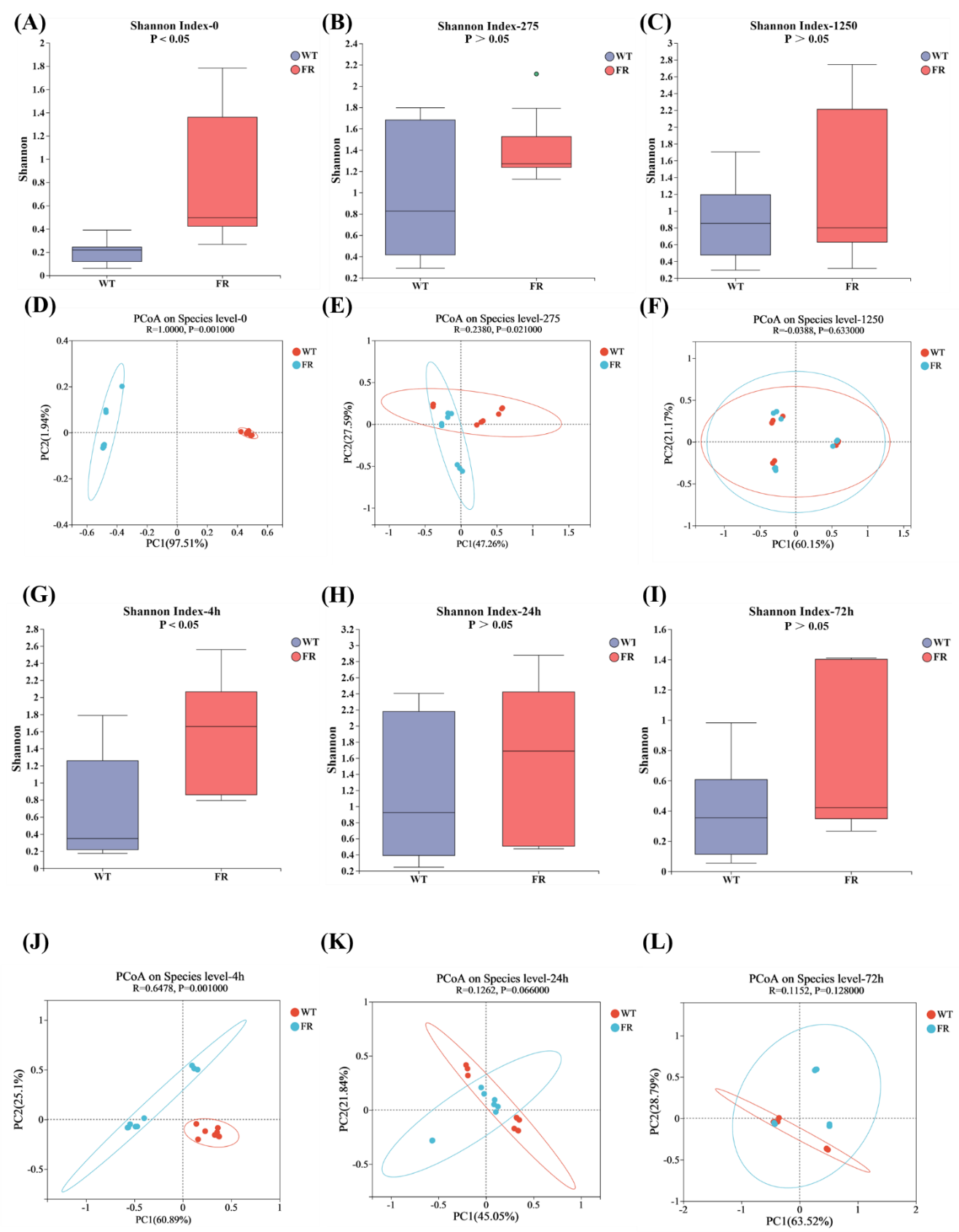


**Supplementary Figure S3.** Overall Shannon indices and PCoA analysis of polymicrobial oral biofilm in the presence of WT/FR. (A-C) The Shannon indices of each concentration of NaF, representing 0 ppm, 275 ppm, 1250 ppm. (G-I) The Shannon indices of each incubating time, standing at 4 h, 24 h, 72 h. (D-F) The PCoA analysis of each concentration of NaF, representing 0 ppm, 275 ppm, 1250 ppm. (G-L) The PCoA analysis of each incubating time, standing at 4 h, 24 h, 72 h.

**Supplementary Table S1.** Primers and probes for qRT-PCR.

| **Primers or probes** | **Sequence (5’-3’)** | **References** |
| --- | --- | --- |
| *S. mutans*-Taqman-f | GCCTACAGCTCAGAGATGCTATTCT | (Zheng et al., 2017) |
| *S. mutans*-Taqman-r | GCCATACACCACTCATGAATTGA |  |
| *S. mutans*-Taqman-probe | TGGAAATGACGGTCGCCGTTATGAA |  |
| Universal bacteria-Taqman-f | CGCTAGTAATCGTGGATCAGAATG | (Zheng et al., 2017) |
| Universal bacteria-Taqman-r | TGTGACGGGCGGTGTGTA |  |
| Universal bacteria-Taqman-probe | CACGGTGAATACGTTCCCGGGC |  |

**Supplementary Table S2.** Probes for Fluorescence in situ hybridization (FISH).

| **Probe name** | | **Sequence (5’-3’)** | **References** |
| --- | --- | --- | --- |
| *S. mutans* | (Alexa fluor 488)-ACTCCAGACTTTCCTGAC | | (Zhu et al., 2021) |
| Universal bacteria | (Alexa Fluor 405)-GCTGCCTCCCGTAGGAGT | | (Zhu et al., 2021) |

**REFERENCES**

Zheng X., He J., Wang L., Zhou S., Peng X., Huang S., et al. (2017). Ecological effect of arginine on oral microbiota. Sci. Rep. 7(1), 7206. doi: 10.1038/s41598-017-07042-w

Zhu J., Liu J., Li Z., Xi R., Li Y., Peng X., et al. (2021) The effects of nonnutritive sweeteners on the cariogenic potential of oral microbiome. *Biomed. Res. Int.* 2021, 9967035.
